# Zinc and chitosan-enhanced β-tricalcium phosphate from calcined fetal bovine bone for mandible reconstruction

**DOI:** 10.3389/fbioe.2024.1355493

**Published:** 2024-09-27

**Authors:** Jianye Zhou, Rui Ma, Wen Shi, Shennan Lei, Xiaohui Zhang, Nan Jiang, Yongsheng Lin, Zhiqiang Li, Min Nie

**Affiliations:** ^1^ Key Laboratory of Oral Diseases of Gansu Province, Key Laboratory of Stomatology of State Ethnic Affairs Commission, Northwest Minzu University, Lanzhou, Gansu, China; ^2^ Institute of Applied Ecology, Chinese Academy of Sciences, Shenyang, Liaoning, China; ^3^ Department of Periodontics, Affiliated Stomatology Hospital of Guangzhou Medical University, Guangdong Engineering Research Center of Oral Restoration and Reconstruction, Guangzhou Key Laboratory of Basic and Applied Research of Oral Regenerative Medicine, Guangzhou, China

**Keywords:** chitosan, zinc, calcined fetal bovine bone, beta-tricalcium phosphate, biocompatibility, physicochemical properties, osteogenic ability

## Abstract

**Background:**

Mandibular defects pose significant challenges in reconstructive surgery, and scaffold materials are increasingly recognized for their potential to address these challenges. Among various scaffold materials, Beta-tricalcium phosphate (β-TCP) is noted for its exceptional osteogenic properties. However, improvements in its biodegradation rate and mechanical strength are essential for optimal performance.

**Methods:**

In this study, we developed a novel β-TCP-based scaffold, CFBB, by calcining fetal bovine cancellous bone. To enhance its properties, we modified CFBB with Chitosan (CS) and Zinc (Zn), creating three additional scaffold materials: CFBB/CS, CFBB/Zn^2+^, and CFBB/Zn^2+^/CS. We conducted comprehensive assessments of their physicochemical and morphological properties, degradation rates, biocompatibility, osteogenic ability, new bone formation, and neovascularization both *in vitro* and *in vivo*.

**Results:**

Our findings revealed that all four materials were biocompatible and safe for use. The modifications with CS and Zn^2+^ significantly improved the mechanical strength, osteogenic, and angiogenic properties of CFBB, while concurrently decelerating its resorption rate. Among the tested materials, CFBB/Zn^2+^/CS demonstrated superior performance in promoting bone regeneration and vascularization, making it a particularly promising candidate for mandibular reconstruction.

**Conclusion:**

The CFBB/Zn^2+^/CS scaffold material, with its enhanced mechanical, osteogenic, and angiogenic properties, and a controlled resorption rate, emerges as a highly effective alternative for the repair of oral mandible defects. This study underscores the potential of combining multiple bioactive agents in scaffold materials to improve their functionality for specific clinical applications in bone tissue engineering.

## 1 Introduction

Repairing mandibular defects arising from tumors, inflammation, and dental bone problems (Maruf et al., 2022) is crucial, as it significantly contributes to maintaining facial aesthetics and essential functions like chewing and articulation. The blood supply to the mandible is less abundant than the maxillary bone, primarily depending on the inferior alveolar artery’s branches for circulation. Consequently, in mandibular repair, employing multi-space stent materials that facilitate neovascularization is of greater importance (Maruf et al., 2023). Though there are numerous advanced methods in processing technology for artificial bone scaffold materials, existing artificial technologies struggle to replicate the characteristics of natural bone scaffolds and element composition, such as their inherent connectivity, mixed sizing, and gradient arrangement (Maruf et al., 2023; Han et al., 2022; Won et al., 2022).

Due to its porous structure, similar to human cancellous bone, and its excellent osteogenic effects, bovine bone stands out in the research of natural bone scaffold materials (Monmaturapoj et al., 2022). Especially in our preliminary studies, it was found that calcined fetal bovine bone has a superior porous structure and osteogenic effect compared to calcined adult bovine bone (Jianye et al., 2017). It also caters to the excellent porous structure required to repair bone and realizes the utilization of bio-waste yielded from the increasing consumption of beef and fetal bovine serum-based products (Han et al., 2022; Amid et al., 2021), avoiding the disadvantages of expensive materials and complex processing.

In calcined natural bovine bone scaffold materials, hydroxyapatites (HA) and beta-calcium phosphate tribasic (β-TCP) are the primary background materials (Monmaturapoj et al., 2022). The slow degradation rate of HA can result in the persistence of HA-based calcined natural bovine bone materials, which may become a source of infection or obstruct the successful integration of implanted objects (Monmaturapoj et al., 2022). In contrast, bone substitutes that include β-TCP as the main component have better osteogenic ability than HA and are easily replaced by new bone (Yuan et al., 2010; Bohner et al., 2020; Bohner et al., 2017). However, the fast absorption rate of β-TCP and poorer mechanical properties limit their use, particularly in load-bearing sites (Bad et al., 2014).

Surface modifications play a crucial role in enhancing the characteristics of various materials (Lapshin et al., 2010). In addition, for clinical application, a qualified bone substitute must have good biocompatibility, making it essential to select surface modification materials that are effective and safe (Jodati et al., 2020). The safety of β-TCP is well-established (Hossain et al., 2022), so it needs to be emphasized that its surface modification material should also be secure and effective.

Chitosan (Cs) is a natural alkaline polysaccharide with a positive charge, which can promote and accelerate bone growth (Elkholy et al., 2018; Yin et al., 2021) and has a broad spectrum of antibacterial activity, inhibiting the growth of various fungi, bacteria, and yeast (Kong et al., 2010). However, due to their low mechanical strength and poor structural stability, Cs are often combined with other materials to form ideal bone scaffolds (Venkatesan and Kim, 2010; Singh et al., 2019). Zinc (Zn^2+^) is another mature surface modifier that can promote bone formation by regulating the synthesis and activity of bone-specific transcription factors (Xue et al., 2008) and improving the mechanical strength of β-TCP scaffolds (Bandyopadhyay et al., 2007). Furthermore, Zn^2+^ exhibits moderate activity in regulating angiogenesis (Xue et al., 2008; Wang et al., 2022) and has a notable antibacterial effect in bone scaffolds (Deng et al., 2022). Therefore, it is worth exploring whether Zn^2+^ can be combined with Cs to conduct surface modification on β-TCP-based bone scaffolds, thus improving their mechanical strength and osteogenic ability.

This study aimed to modify fetal bovine bone scaffold materials prepared by calcinating ammonium dihydrogen phosphate with Zn^2+^ and CS. The biocompatibility, mechanical strength, and comprehensive bone repair ability were explored *in vivo* and *in vitro* to provide an experimental basis for their clinical application and promotion.

## 2 Materials and methods

### 2.1 Sample preparation

Aborted fetal calves were purchased from a cattle farm in Gannan Tibetan Autonomous Prefecture, Gansu Province. Fresh femurs from fetal calves were boiled with distilled water at 95°C for 10 h to remove soft tissue. The cancellous bone of the head of the femurs was cut into cuboid bone blocks of 5 mm × 5 mm x 40 mm (for mechanical property detection only) and 5 mm × 5 mm x 10 mm (for all other experiments). A solution of 0.25 mol/L NaOH (Sigma Aldrich (Shanghai) Trading Co., Ltd., Shanghai, China) and 10% H_2_O_2_ was used to remove fat, protein, and other organic matter. The bone blocks were washed with distilled water and dried before being calcined in a muffle furnace at 800°C for 6 h with a heating rate of 5.0°C/min. After natural cooling, the bone blocks were cleaned with ultrasonic waves and dried before being immersed in a 0.50 mol/L NH_4_H_2_PO_4_ (Sigma Aldrich (Shanghai) Trading Co., Ltd., Shanghai, China) solution and sealed for 24 h at room temperature (Ito et al., 2006). The bone blocks were dried again before being slowly heated in a muffle furnace to 1,000°C for 4 h (Pramanik et al., 2015). Then, the β-TCP-based calcined natural fetal bovine bone samples were obtained after natural cooling, and we named it CFBB.

For CFBB/Zn^2+^ samples, the partial CFBB samples were fully immersed in 0.25 M ZnCl (Sigma Aldrich (Shanghai) Trading Co., Ltd., Shanghai, China) solution (with a ratio of 10 g CFBB samples to 50 mL ZnCl) using a negative pressure suction device with evacuation rate of 3.6 m^2^/h and an evacuation pressure of 0.1 Pa at 60°C for 24 h. The samples were then calcined at 1,000°C for 1 h; For CFBB/CS samples, the partial CFBB samples were immersed in a 10 g/L solution of CS-acetic acid (Sigma Aldrich (Shanghai) Trading Co., Ltd., Shanghai, China) (with a ratio of 10 g CFBB to 20 mL CS-acetic acid solution) for 24 h using a negative pressure suction device. The samples were then immersed in a dilute ammonia solution for 12 h to neutralize residual acetic acid. At last, the samples were rinsed with 0.01 mol/L PBS and dried.

Half of the CFBB/Zn^2+^ samples were further treated using the same preparation method as CFBB/CS samples to form CFBB/Zn^2+^/CS samples. Thus, four groups of these scaffold materials formed and named CFBB, CFBB/Zn^2+^, CFBB/CS, and CFBB/Zn^2+^/CS. All samples were autoclave sterilized before use.

### 2.2 Physicochemical and morphological characterization

#### 2.2.1 Surface morphology and elemental composition

The surface morphology was determined using a scanning electron microscope with an energy-dispersive X-ray detector (SEM-EDX) (Carl Zeiss Management Co., LTD., Shanghai, China). Samples were vacuum-dried and sprayed with gold for 60 s for all four groups. The microstructure observation was performed at 40 kV and 150 mA; magnification images were obtained for each sample with 150 and 500-fold. The aperture size of the samples in these images was measured using SEM’s own estimating software.

Content measurement of Zn^2+^ and CS: The content of Zn^2+^ in the CFBB/Zn^2+^ was analyzed by EDX with a scanning voltage of 10 kV and was detected by randomly selecting five points from each sample; The content of CS was measured using the weighing method. Samples from each CS-containing group were chosen randomly, and the weight changes of bone blocks before and after the modification procedure with CS were measured.

#### 2.2.2 Porosity analysis: X-ray micro-computed tomography (Micro-CT) analysis

Samples were randomly selected from the four groups. Micro-CT and CT-analyzer software (Shanghai Rutuo Biotechnology Co., LTD., Shanghai, China) was used to perform 360° scanning of all scaffold materials with a spatial resolution of 7 μm. The X-ray source was set to 70 kVp and 114 mA. The porosity of the material was calculated using the formula: Porosity = (apparent volume - actual volume)/apparent volume ×100%.

#### 2.2.3 Fourier transform infrared spectroscopy (FTIR) characterization

Chemical characterization was performed using the FTIR (China Scientific Equipment Co., Ltd., Beijing, China) with the KBr tablet method. Samples were randomly sampled in each group, dried, and finely ground in an agate mortar. After sifting with a 300-mesh screen, the samples were ground and mixed evenly with KBr at a ratio of 1:100. The mixture was then put into a dry mold, emptied, and pressurized to produce a transparent sheet. The dried samples were ground and mixed evenly with pure KBr powder. The mixture was then placed in a dry mold and pressed into a transparent sheet. Samples from each group were tested at room temperature with an indoor relative humidity of less than 70%. We performed three scans for each group of samples.

#### 2.2.4 Structural properties: X-ray diffraction (XRD) analysis

Samples were randomly selected from each group. The samples were ground in an agate mortar and screened with a 300-mesh screen to produce as even a sample powder as possible to obtain more accurate results. About 0.5 g of sample powder was taken and placed on a glass sample rack. The positive pressure method was used to sample. XRD patterns were obtained using an X-ray diffractometer (Brooke (Beijing) Technology Co., LTD., Beijing, China), which operated at 40 kV and 150 mA with Cu Kɑ radiation (λ = 1.5406 Å). The diffractograms were collected from 10° to 70° on a 2*θ* scale, with a step size of 0.02° and a scanning speed of 10°/min. Mapping and analysis were conducted using MDI Jade 6.5 software with the standard cards of HA [Ca_5_(PO4)_3_(OH), JCPDF No. 09-0432] and CFBB [Ca_3_(PO_4_)_2_, CFBB, JCPDF No. 09-0160].

#### 2.2.5 Compressive and flexural strength measurements

Compressive and flexural strength measurements were tested using a universal material testing machine (UMTM) (Instron, Illinois Tool Works Inc. London, America). Samples were randomly selected from each of the four groups to test the compressive (5 mm × 5 mm × 40 mm) and flexural strength (5 mm × 5 mm × 40 mm). The loading speed was kept constant at 0.5 mm/min with the 200 N load cell, and the three measurements were averaged. The flexural test was conducted to evaluate the compressive and flexural strength of the materials. The load plate was securely installed on the testing machine to ensure stability during the test. The loading speed of the testing machine was set to 2 × 10^2^ N/s and maintained consistently throughout the testing process. Each group of materials underwent compressive and flexural strength testing. During the tests, changes in the samples were closely monitored, and the pressure value was accurately read and recorded at the point of catastrophic failure. To ensure the accuracy and reliability of the test results, each material was tested ten times.

### 2.3 Cell line experiments

#### 2.3.1 Cell culture

Mouse osteoblast-like cells (MC3T3-E1) (Wuhan Sios Biotechnology Co. LTD., Wuhan, China) were cultured in DMEM/F12 medium containing 10% fetal bovine serum (FBS) (Thermo Fisher Scientific Inc (Shanghai), Shanghai, China) under 5% CO_2_ at 37°C. Samples were randomly selected from each group and immersed in DMEM/F12 at 200 mg/mL for 1 day at 37°C to get extract A. For extract B, samples were randomly selected from each group and immersed in normal saline at 200 mg/mL for 1 day at 37°C. The extracts were sterilized using 0.22 μm filters before use.

#### 2.3.2 MTT assay

200 μL of MC3T3-E1 cells at 5×10^4^ cells/mL were seeded in 96-well plates and incubated for 24 h. The supernatant of each group was replaced with medium extract A. The cells exposed to 200 μL of DMEM/F12 medium served as the control group. The cytotoxicity of samples in each group was evaluated by the 3-(4,5-dimethylthiazol-2-yl)-2,5-diphenyl-2H-tetrazolium bromide (MTT) assay at 48 and 72 h.

#### 2.3.3 Cell adhesion

1 mL MC3T3-E1 cell suspension containing 1×10^5^ cells was seeded onto scaffolds of the same size for each group. The cells and scaffolds were cultured at 37°C with 5% CO_2_ for 4 and 7 days. The medium was changed every 48 h. Afterward, the samples were rinsed with PBS, fixed using 4% glutaraldehyde (Sigma Aldrich (Shanghai) Trading Co., Ltd., Shanghai, China), and dehydrated with ether for 15 min, after which the ether was removed. The SEM observations were conducted as described in 2.2.1.

#### 2.3.4 RNA extraction and real-time quantitative PCR (RT-qPCR) assay

Samples in each group were placed into 24-well plates and seeded with 1 mL of MC3T3-E1 (5×10^4^ cells/mL). Wells with only MC3T3-E1 cells served as the blank control. After incubation for 14 days, the cells of all groups were collected after trypsin digestion and centrifugation. The total RNA was extracted and reversed transcribed into complementary DNA (cDNA) with reverse transcriptase using the TaKaRa MiniBEST Universal RNA Extraction Kit and PrimeScript™ RT reagent Kit with gDNA Eraser (Takara Biomedical Technology (Beijing) Co., Ltd., Beijing, China) according to the manufacturer’s protocols. The expression of osteopontin (OPN), osteocalcin (OCN), and collagen type I (COL I) were tested by RT-qPCR, which was performed with ABI 7500 Thermal cycler (Huake Jianlian Gene Technology (Beijing) Co., LTD., Beijing, China). All samples were tested in triplicate and repeated three times each. The PCR primers are shown in Table 1.

**TABLE 1:** Primer sequence list.

Gene	Upstream primer	Downstream primer
GAPDH	5′AGG​TCG​GTG​TGA​ACG​GAT​TTG 3′	5′GGG​GTC​GTT​GAT​GGC​AAC​A 3′
β-actin	5′ACC​GAC​TAC​CTC​ATG​AAG​ATC​CT3′	5′TCG​TTG​CCG​ATG​GTG​ATG​A 3′
COL I	5′CAA​CAG​CAG​GTT​CAC​TTA​CAC​T 3′	5′CAA​GGA​AGG​GCA​AAC​GAG​AT 3′
OPN	5′ACC​AAG​GAA​CAA​TCA​CCA​CCA​T3′	5′TAG​CAT​TCT​GCG​GTG​TTA​GGA​G 3′
OCN	5′GAA​ACC​GAA​GAG​GAA​GTA​GTG​G3′	5′AAA​GAA​GTG​GCA​GGA​GGA​GTC 3′

#### 2.3.5 ALP assay

Samples in each group were placed into 24-well plates with cells as described in 2.3.4. After incubation for 24 h, the cells of all groups were collected after trypsin digestion, centrifugation, and treatment. Then, alkaline phosphatase (ALP) activity was measured with an ALP test kit (Shanghai Langton Biotechnology Co., Ltd., Shanghai, China) according to the manufacturer’s instructions. All samples were tested in triplicate and repeated three times each.

### 2.4 Hemolysis test

1 mL of blood was collected from the auricular vein of a New Zealand rabbit (Lanzhou Veterinary Research Institute, Lanzhou, China) and mixed with heparin in a tube (Beijing Biolaibo Technology Co., LTD., Beijing, China). The blood sample was diluted twice with normal saline before use. The hemolysis experiment was conducted using the extract B of the four groups. The positive control group was treated with distilled water, and the negative control group was treated with normal saline. The optical density (OD) was measured using a UV spectrophotometer (Hangzhou Junsheng Scientific Equipment Co., LTD., Hangzhou, China) to calculate the hemolysis rate. The hemolysis rate was determined using the following equation: Hemolysis rate (%) = (experimental group OD - negative control OD)/(positive control OD - negative control OD) ×100%.

### 2.5 Animal experiments

#### 2.5.1 Systemic acute toxicity

Thirty healthy Kunming mice (20–22 g in weight, half male and half female) (Lanzhou Veterinary Research Institute, Lanzhou, China) were randomly divided into the control and four test groups (n = 6). The control group was injected with normal saline intraperitoneally, and the other groups were injected intraperitoneally with the corresponding extract B from 2.3.1. The general state of mice in each group was observed at 4, 24, 48, and 72 h after injection, including their activity, mental state, respiration, gait, the number of dead animals, and toxicity manifestations. Mice were sacrificed 1 week after injection, and the pathological changes in the kidney, heart, and liver were observed by HE staining.

#### 2.5.2 Establishment of animal models

Forty-eight New Zealand rabbits (half male and half female, weighing 1.8–2.8 kg) (Lanzhou Veterinary Research Institute, Lanzhou, China) were randomly assigned to the four groups. The bilateral mandibular foramen was selected as the anterior insertion of the surgical area. In contrast, the anterior notch of the mandibular angle was used as the posterior insertion of the surgical site. A box-shaped artificial bone defect measuring 5 mm × 5 mm × 10 mm was prepared using a high-speed ball drill about 1 cm from the mandible’s lower edge toward the surgical area’s buccal side. The four groups of samples were then filled and sutured in layers. The CFBB and CFBB/Zn^2+^ samples were implanted into the same rabbit’s bilateral mandibular body bone defects, while the samples of CFBB/CS and CFBB/Zn^2+^/CS were also implanted into the same rabbit. All surgical procedures were performed in the GMP animal laboratory under strict aseptic techniques, debridement, and hemostasis (Supplementary Figure S1). The animal was sacrificed with an overdose of anesthesia at 4 weeks, 8 weeks, and 12 weeks after surgical (n = 4, 2 males and 2 females). Each observation point yielded experimental samples obtained using anatomical methods appropriate for rabbits that had been euthanized.

##### 2.5.2.1 Detection of new bone formation volume, material degradation rate, and neovascularization area

Specimens were obtained using a wire saw approximately 2 mm outside the perimeter of the bone graft site and fixed with 4% paraformaldehyde. The specimens were then scanned in micro-CT (Shanghai Rutuo Biotechnology Co., LTD., Shanghai, China) to perform 360° scanning with a spatial resolution of 7 μm. The X-ray source was set to 70 kVp and 114 mA. The CT-analyzer software (Shanghai Rutuo Biotechnology Co., LTD., Shanghai, China) was used to reconstruct and analyze the volume of residual material of specimens. The volume of residual material (%) was calculated as the volume of residual material in the bone defect area divided by the volume of implanted material multiplied by 100%. Simultaneously, a preliminary rough assessment was made to determine the location and volume of the new bone.

##### 2.5.2.2 Histological and immunohistochemistry

The specimens were decalcified using the micro wax-EDTA rapid decalcification technique for 1 week, then routinely dehydrated, immersed in wax, and embedded. A series of consecutive 5 μm thick sections were obtained from the center of the cuboidal bone defect, from buccal to lingual, and from mesial to distal directions. Ten sections were prepared for each sample, and 5 sections were randomly selected for Masson’s trichrome staining to evaluate the volume and maturity of new bone in the bone defect area. The total area of new bone in the bone defect area in each image was calculated using Image-ProPlus 6.0. The other 5 sections were immunohistochemically stained using the SP method. The primary antibody used was anti-endothelial (PAL-Eab8086). The secondary antibody used was biotin-labeled goat anti-mouse Ig G. Three fields (1mm2/field) were selected for each slice under a 100× microscope. The total area of neovascularization in each image was calculated using Image-ProPlus 6.0.

### 2.6 Statistical analysis

The statistical analysis was carried out using the Statistical Package for Social Science (SPSS 23.0, IBM Corporation, Armonk, NY, USA). Pairwise comparison between groups was conducted using the independent t-test, and three or more groups were compared using one-way ANOVA. *P* < 0.05 were considered statistically significant.

## 3 Results

### 3.1 Physicochemical and morphological characterization

**FIGURE 1:**
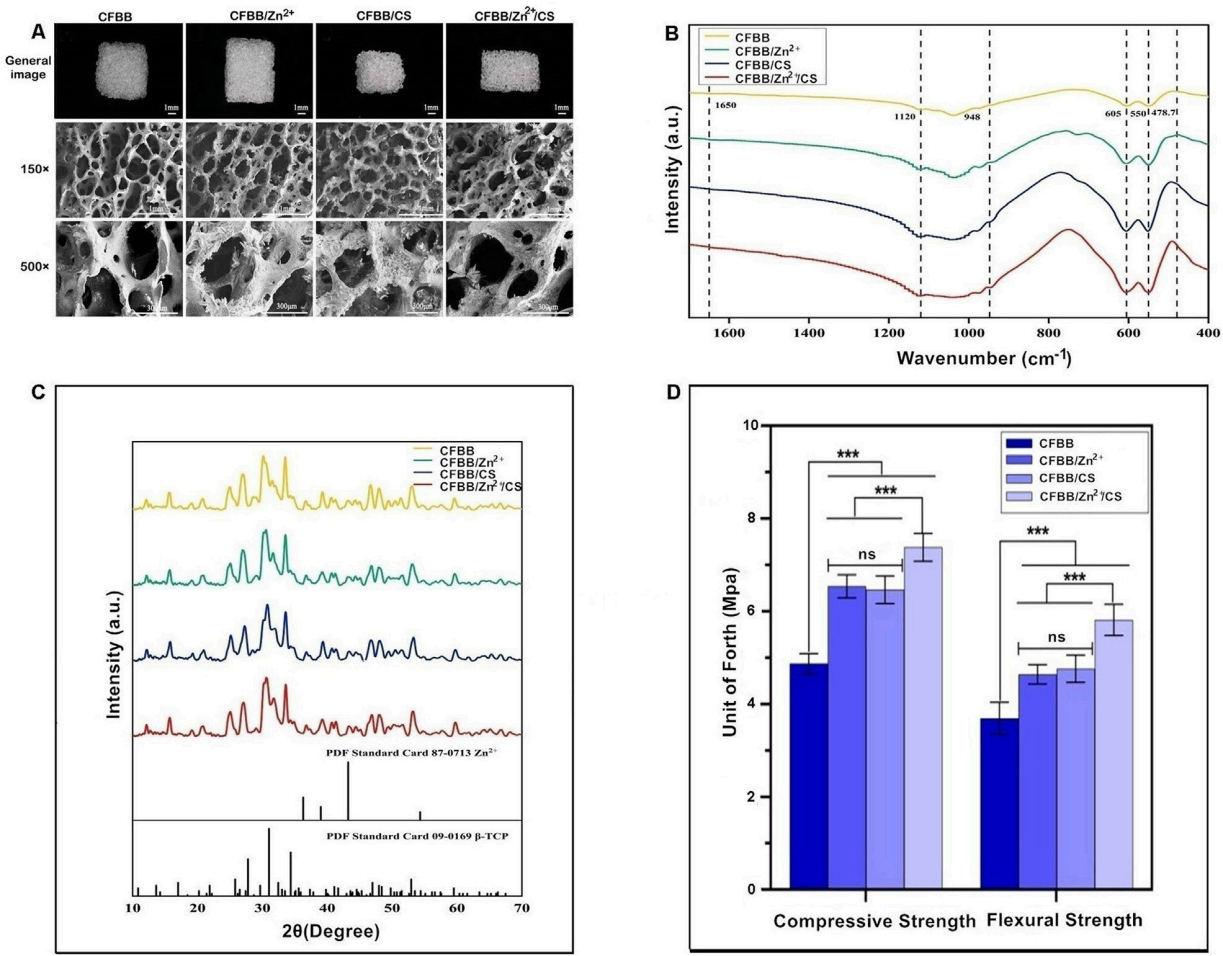
Physicochemical and morphological characterization. **(A)**, General appearance and scanning electron microscopy (SEM) images; **(B)**, FTIR analysis, unit of forth (Mpa): The units of compressive and flexural strength used in this study are in MPa; **(C)**, XRD analysis; **(D)**, Compressive and flexural strength. *** indicates *p* < 0.001, and ns indicates no statistical difference.

The general appearance and microstructure of the samples retained the bone trabeculae and pores of natural cancellous bone, which were connected through pores (Figure 1A). The morphology of CFBB was unchanged upon loading of Zn^2+^, but the addition of CS formed a continuous organic film on the surface of calcined bone.

The FTIR analysis of samples revealed characteristic frequencies of CFBB (Figure 1B). The bending vibration peak of PO4^3-^ was observed at 550 cm^-1^-605 cm^-1^, while the stretching vibration peak of PO4^3-^ was observed at 948 cm-1-1,120 cm-1, indicating that β-TCP is the main component of the prepared samples. The characteristic peak of ZnCL was observed at 478.7 cm^-1^ in the CFBB/Zn^2+^. The CFBB/CS and CFBB/Zn^2+^/CS, the CO = stretched acetylamino peak (which indicates CS loading) was observed at 1,650 cm^-1^ (Tan et al., 2020).

XRD analysis demonstrated the presence of the CFBB phase and the maximum diffraction peak at 31° in all samples (Figure 1C). The maximum diffraction peak of Zn^2+^ was observed at 42° in the CFBB/Zn^2+^ and CFBB/Zn^2+^/CS. However, due to the low content of Zn^2+^ (0.32%), the diffraction peak was not obvious. The diffraction peaks of CS were observed at 10° and 20°. Due to the crystallization characteristics caused by the hydrogen bonding of amino hydroxyl molecules, the diffraction peaks were relatively smooth, making it difficult to observe in the CFBB/CS and CFBB/Zn^2+^/CS.

According to the compressive strength and flexural strength, CFBB/Zn^2+^, CFBB/CS, and CFBB/Zn^2+^/CS were all significantly higher than CFBB (*P* < 0.001) (Figure 1D); Moreover, the CFBB/Zn^2+^/CS was much higher than the CFBB/Zn^2+^ and CFBB/CS (*P* < 0.001); There was no significant difference between the CFBB/Zn^2+^ and CFBB/CS groups.

In addition, the content of Zn^2+^ in CFBB/Zn^2+^ measured by EDAX was 0.32 wt. As CFBB/Zn^2+^/CS was prepared based on CFBB/Zn^2+^ and could not be measured by EDAX, the content of Zn^2+^ in CFBB/Zn^2+^/CS was considered to be lower than 0.32 wt; After calculation, the mass ratios of CS in CFBB/CS and CFBB/Zn^2+^/CS were 0.7% and 0.71%, respectively; About the porosity, there were no significant differences in pore size and porosity between the four groups, with all values for CFBB being 193.28 ± 42.82 (μm) and 70.61 ± 0.34 (%), respectively.

### 3.2 Biocompatibility evaluation *in vitro* and *in vivo*

**FIGURE 2:**
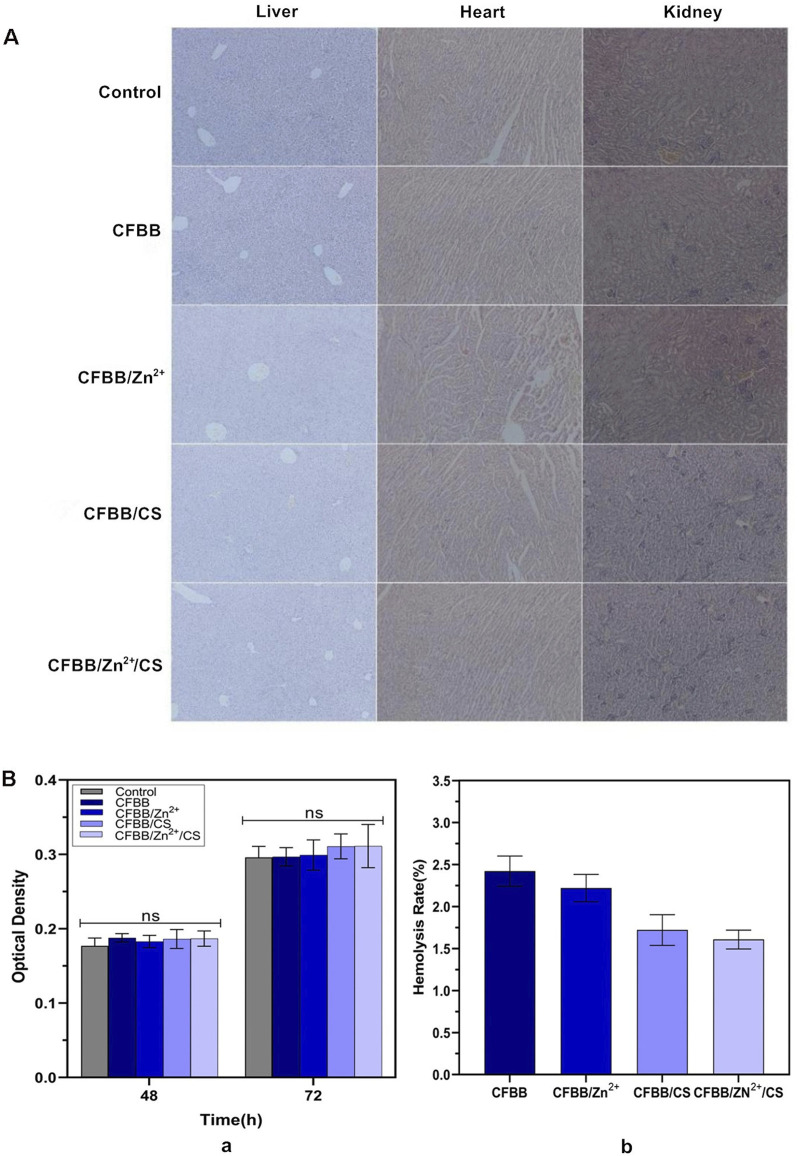
Biocompatibility evaluation. **(A)**, HE sections of liver, heart, and kidney (100×); **(B)** (a), MTT assay; **(B)** (b), hemolysis test.

The biocompatibility of the prepared materials was evaluated through systemic acute toxicity verification, cytotoxicity, and hemolysis tests. The systemic acute toxicity test results indicated that during the 1-week observation period, all mice in each group displayed normal activities, consumed food and water, and did not exhibit any toxic reactions or mortality. Moreover, the average body weight of mice in each group increased by approximately 1g before and after the experiment. Tissue sections subjected to HE staining showed that the heart, liver, and kidney tissues were normal (Figure 2A), and we conducted preliminary tests on the tissue sections. Based on the acute toxic dose evaluation, all samples were classified as non-toxic.

The MTT assay revealed no significant differences in the relative growth rate (RGR) between the four groups and the blank control group (normal saline) at 48 h and 72 h (*P* > 0.05) (Figure 2B(a)). Thus, the cytotoxicity of materials in all four groups was assessed as level 0 (Yang et al., 2018).

The hemolysis test results demonstrated that the average hemolysis rate of the four groups in fresh rabbit blood was less than 5% (Figure 2B(b)), within the allowable range (ASTM International, 2017). These findings all indicate that the safety of each sample was satisfactory.

### 3.3 Cell adhesion *in vitro*

**FIGURE 3:**
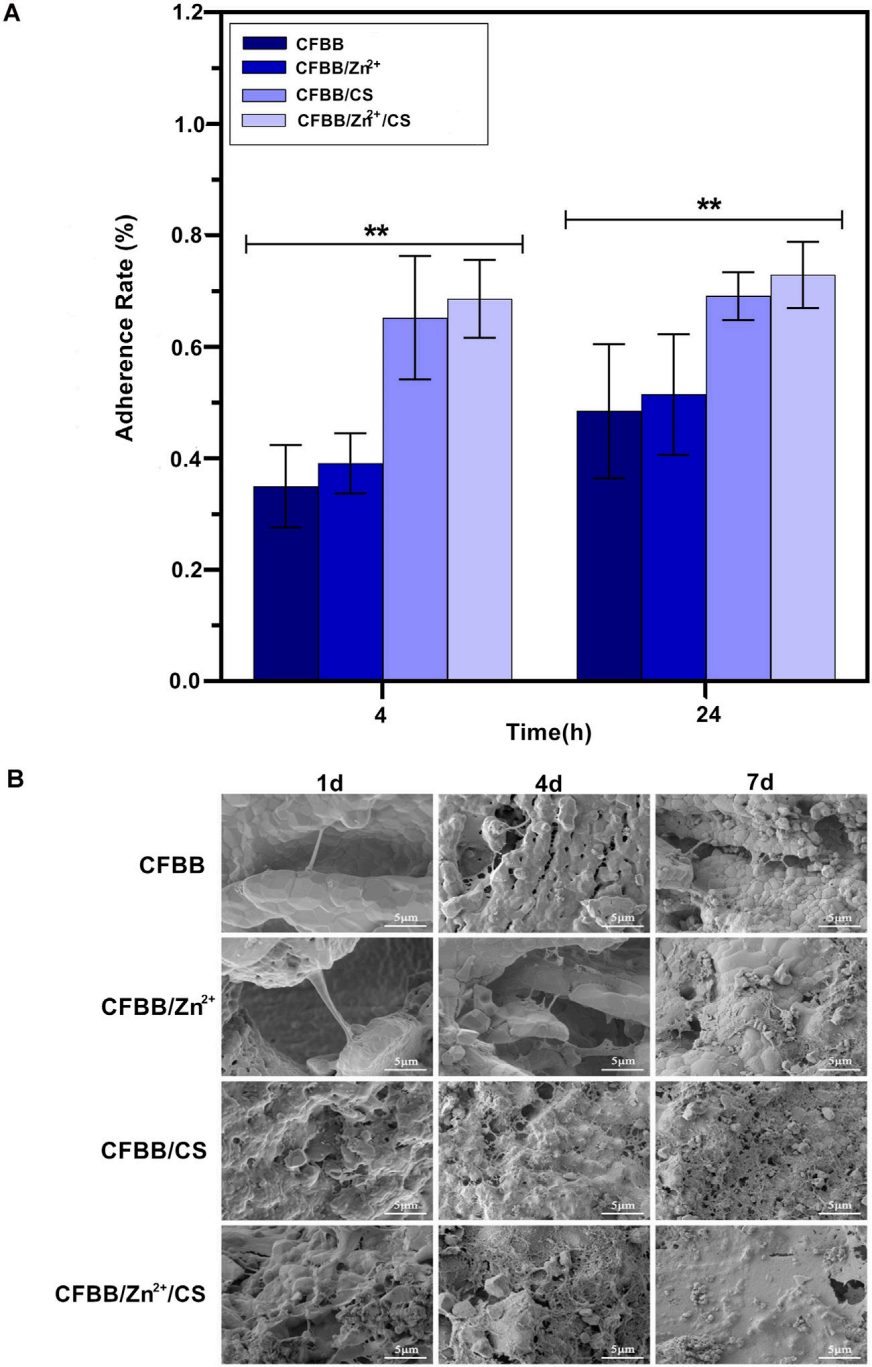
Cell adhesion. **(A)**, Cell adhesion rate; **(B)**, SEM image of cell adhesion. * indicates *p* < 0.01.

The results of the cell adhesion assay showed that surface modifications of CS and Zn^2+^ positively affected scaffold adhesion. The CFBB/Zn^2+^/CS exhibited the highest rate of cell adhesion among the four groups (Figure 3A) (*P* < 0.01). After 4 days of inoculation, the SEM images revealed a significant increase in the number of cells on the scaffold. Many cells extended their pseudopodia and surrounded the scaffold. By day 7, numerous cells and collagen fibers covered the scaffold and formed sheets, indicating cell proliferation and integration with the scaffold (Figure 3B). These findings suggest that the surface modifications of CS and Zn^2+^ can promote cell adhesion and proliferation on bone scaffolds and may have applications in promoting osteogenesis.

### 3.4 Osteogenic ability test *in vitro* and *in vivo*

**FIGURE 4:**
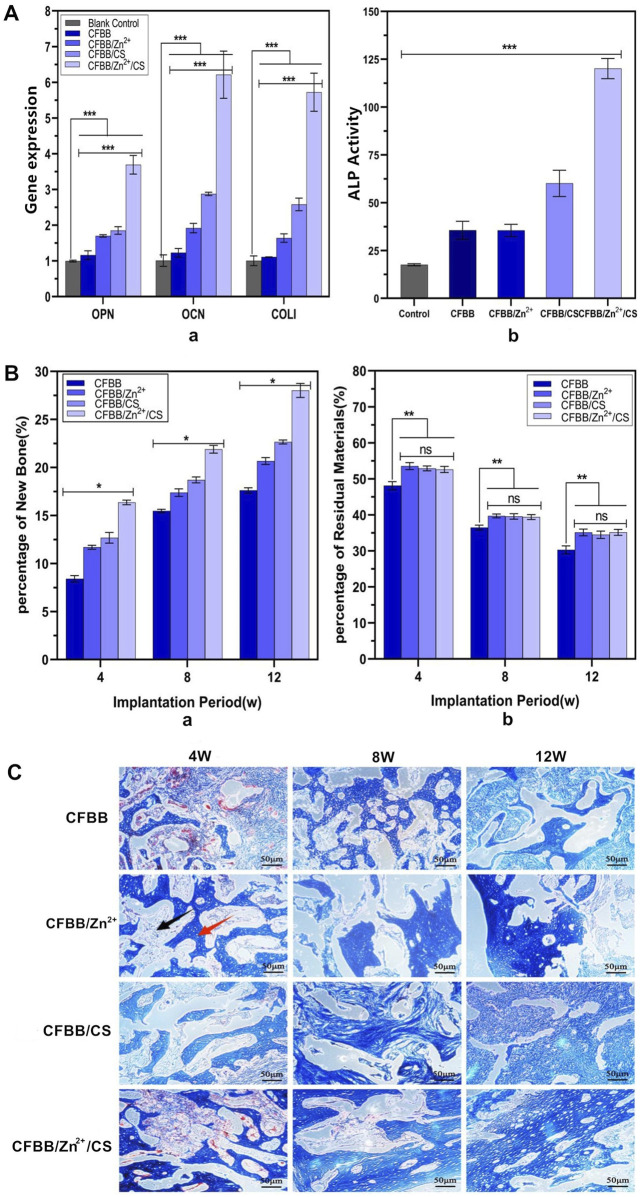
Osteogenic ability. **(A)** (a) expression of OPN, OCN, and COL Ⅰ; **(A)** (b) ALP activity. Miro-CT analysis of new bone formation volume **(B)** (a) and material graduation rate **(B)** (b); (**C)**, Masson staining of new bone tissue (100X). ** indicates *p* < 0.01, * indicates *p* < 0.05, and ns indicates no statistical differences.

The osteogenic induction ability of the samples was evaluated through the expressions of OPN, OCN, and COL I genes and ALP activity *in vitro*. Figure 4A (a) shows the expressions of OPN, OCN, and COL I genes, and Figure 4A (b) shows the ALP activity between the black control group and the four material groups. These indexes’ expressions were significantly lower in the black control group than in the material groups (*p* < 0.001). The index of the four groups showed a decreasing trend as follows: CFBB/Zn^2+^/CS > CFBB/CS > CFBB/Zn^2+^ > CFBB (*p* < 0.001). These results suggest that CS and Zn^2+^ have separate roles in promoting osteoblast induction and may synergistically promote osteogenesis.

The repair ability of the prepared materials was evaluated *in vivo* through micro-CT analysis and Masson staining. Figure 4B(a) illustrates the relationship among the new bone formation volume of the four groups at 4W, 8W, and 12W. The decreasing order of the volume was CFBB/Zn^2+^/CS > CFBB/CS > CFBB/Zn^2+^ > CFBB (*p* < 0.05).

Figure 4B(b) compares the percentage of residual material at 4W, 8W, and 12W among the four groups. The absorption rate of CFBB was lower than that of CFBB/Zn2+, CFBB/CS, and CFBB/Zn^2+^/CS (*p* < 0.01), respectively. The last three groups had no significant difference (*p* > 0.05). These findings suggest that CFBB/Zn^2+^/CS, CFBB/Zn^2+^, and CFBB/CS could slow down the absorption rate of CFBB.

The repair ability of the materials was confirmed by Masson staining (Figure 4C), which revealed a gradual increase in the amount and maturity of new bone in each group over time. These results suggest that CS and Zn^2+^ may synergistically promote osteogenesis and their roles in promoting osteoblast induction.

### 3.5 Area of neovascularization

**FIGURE 5:**
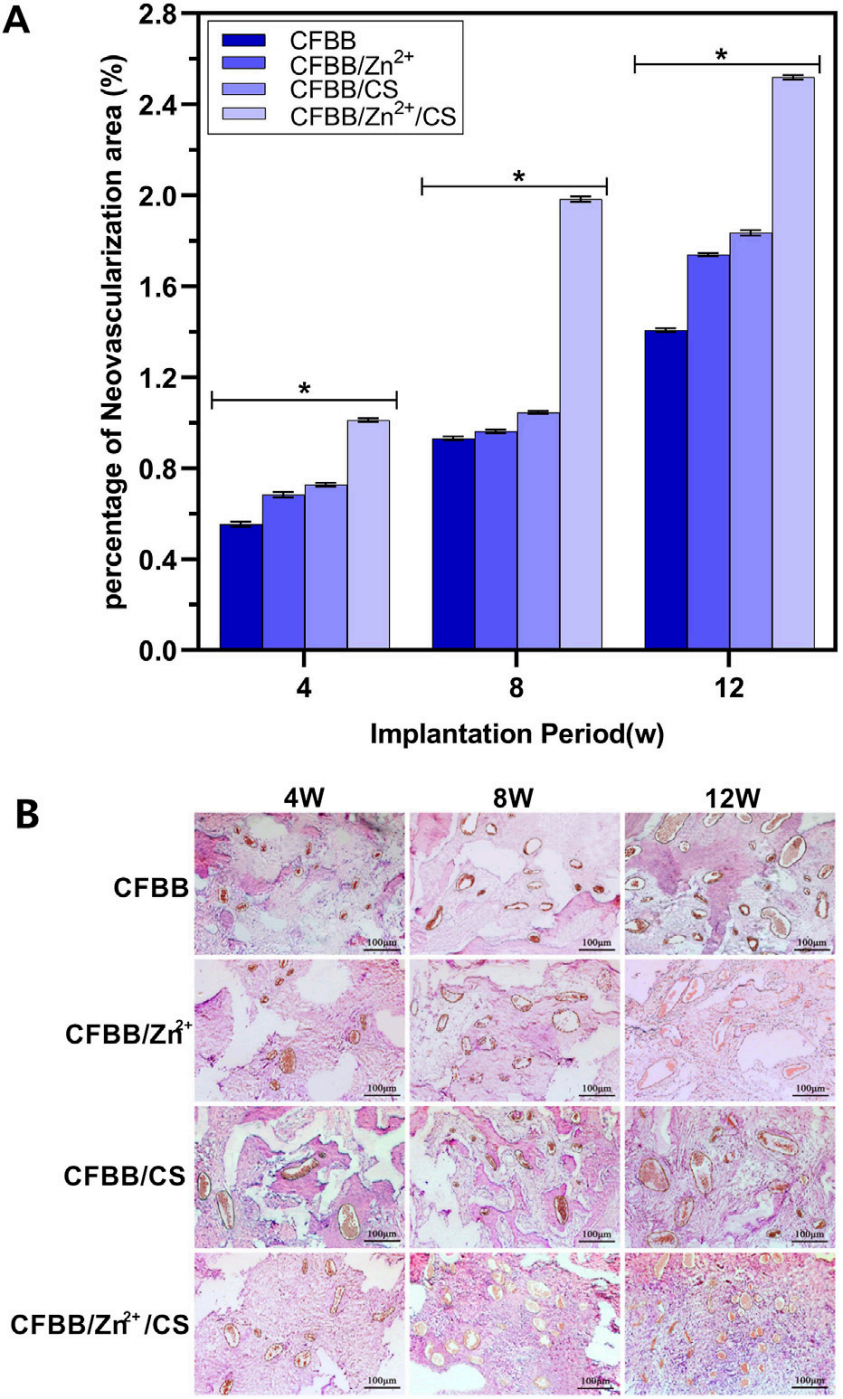
**(A)**, Percentage of new blood vessels; **(B)**, Immunohistochemical staining (100X). * Indicates *p* < 0.05.

Figure 5A shows that the area of neovascularization belonging to the four groups of materials had a declining trend over time (4W, 8W, and 12W). The order of decreasing trend was CFBB/Zn^2+^/CS > CFBB/CS > CFBB/Zn^2+^ > CFBB (*P* < 0.05). The results of immunohistochemical staining (Figure 5B) were consistent with the above findings, as the number and area of new blood vessels per unit area increased in each group with the extension of the implantation cycle.

## 4 Discussion

The osteogenesis effect of bone scaffold materials often differs *in vitro* and *in vivo* due to various uncertain factors (Karageorgiou and Kaplan, 2005; Wu et al., 2016). Therefore, we conducted relatively comprehensive experiments to verify the surface modification effects of Zn^2+^ and chitosan CS on the CFBB *in vitro* and *in vivo*. Our results demonstrated that all materials we studied were deemed safe. Combining with CS or Zn^2+^ significantly improved the mechanical, osteogenic, and angiogenic properties while slowing down the resorption rate of CFBB. This may be due to the synergistic enhancement of scaffold osteoinductivity by their surface chemistry and physical effects (Yu et al., 2018).

Zn^2+^ and CS possess effective mechanisms for inducing osteogenesis in terms of biochemistry. Zn^2+^ promotes the osteogenesis of mesenchymal stem cells (MSCs) through the ZIP family of Zrt- and Irt-like proteins (Bin et al., 2018; Sharma and Merz, 2021). Specifically, Zn^2+^ can activate the PI3K-Akt signaling pathway to promote the expression of BMP-2, Runx-2, OPN, OCN, and ALP, thereby promoting the bone activity of bone scaffold materials (Deng et al., 2022; Yusa et al., 2011; Hadley et al., 2010). ZnT7, a novel member of the Zn transporter (ZnT) family, can encourage the transformation of undifferentiated MSCs into osteoblasts by regulating two essential signaling pathways in osteogenesis induction, Wnt and ERK (Liu et al., 2013).

The strong adhesion ability of CS is the most likely mechanism behind its promotion of osteogenesis (Li and Bai, 2005). CS forms a layer of chitosan film on the surface of the bone scaffold material, which effectively improves the cell adhesion rate of the material. After being modified with CS solution, the surface of CFBB provides recognition and adsorption sites for cells, promotes cell adhesion media, and reduces cell loss rate, thus facilitating cell reproduction. CS not only encourages the expression of several osteogenic factors (OPN, OCN, COL I, and ALP) in this study but also affects the osteogenesis of MSCs by stimulating the expression of cytokines such as platelet-derived growth factor (PDGF), fibroblast growth factor (FGF), insulin-like growth factor (IGF), and tumor growth factor β (TGF-β) (Beringer et al., 2015; Tan et al., 2014). Furthermore, CS is a powerful chelating agent (Feng et al., 2021) and can easily form complexes with heavy metals and transition metals (Ding et al., 2007). When Zn^2+^ and CS were used to form a double finishing material in our study, CS-Zn^2+^ complexes, possibly formed by Zn^2+^ with the amino and hydroxyl groups of CS, were formed on the surface of CFBB. As expected, CFBB/Zn^2+^/CS showed even better combined osteogenic induction and neovascularization of bone scaffolds than Zn^2+^ and CS alone.

The mechanical factors significantly impact bone degradation, absorption, and destruction. Different mechanical factors can lead to other biological behaviors of bone (Giorgio et al., 2021). They are related to bone conduction during bone repair, providing a necessary environment for physiological osteogenic activities (Campion et al., 2011; Hing et al., 2007; Hing et al., 2006; Zadpoor, 2015). Zn^2+^ has been shown to increase the densification and hardness of TCP (Bandyopadhyay et al., 2007) and improve the mechanical properties of CFBB scaffold (Feng et al., 2014), thereby enhancing its osteogenic ability. Our results confirm that Zn^2+^ strengthens the compressive and flexural strength of CFBB. Similarly, the film formed by CS on the surface of CFBB also enhances its mechanical properties. At the same time, Cs-Zn^2+^ complexes reflect the mutual benefits of the two materials in terms of their mechanical properties.

Appropriate compressive strength is essential for cell differentiation and growth (Klincumhom et al., 2022). However, it is important to note that the index is not the bigger, the better. It should be consistent with the compressive strength of the human mandible and other defect sites to avoid the formation of a bone stress barrier (Klincumhom et al., 2022; Holzwarth and Ma, 2011). The compressive strength of human trabecular bone typically ranges from 2 to 45 MPa (Giorgio et al., 2021). In our experiment, the compressive strength of the four groups of materials ranges from 4.84 to 7.9 MPa. In addition, Previous studies have shown that the pore size of bone scaffold materials in the 40–500 μm significantly impacts bone repair ability. Pores around 100 μm facilitate the growth of fibers and small, round blood cells, rapid cell adhesion, and cartilage formation, while pores above 300 μm are favorable for direct osteogenesis (Akay et al., 2004; Kuboki et al., 2001; Kuboki et al., 2002). This study used calcined natural fetal beef trabecular bone as the primary bone scaffold material. It has a mixed pore size with a median of 193.28 and a range of 42.82 μm, which is suitable for osteogenesis and bone repair. Its porosity of 70.61% ± 0.34% also meets the criteria for better osteogenic ability with porosity above 70% (Teixeira et al., 2010). Although the basic requirements can be met, further improvement of the material’s mechanical properties still needs to be investigated because of the higher standards for the material’s mechanical properties used to repair bone defects in load-bearing areas.

Fortunately, adding Zn^2+^ to the material also can reduce the degradation rate of the CFBB, which can solve the problem of the too-fast speed of β-TCP absorption. Zn^2+^ significantly inhibits the differentiation of osteoclast-like cells (Roy et al., 2013), possibly by inhibiting the nuclear factor kappa-β (NF-κβ) protein of osteoclast-like progenitors *in vitro* (Martyniak et al., 2021). Additionally, the Zrt-Irt-like protein mentioned earlier may promote osteogenesis and significantly inhibit osteoclast differentiation while blocking the NF-κB signaling pathway crucial for osteoclast differentiation (Roy et al., 2013). Similarly, adding other polymers, nanoparticles, or other materials to the CS matrix may also affect controlling degradation kinetics (Saravanan et al., 2016). For instance, the complex formed by Ag ions with CS may have very few free reactive functional groups, reducing hydration on contact with liquid and thus reducing degradation rate (Saravanan et al., 2011).

Ideally, bone scaffold materials should be absorbed at a rate similar to new bone formation. However, the degradation of heterogeneous materials and their biocompatibility is contradictory (Hannink and Arts, 2011). On the one hand, the material must be fully compatible with the host and cannot cause any immune tissue reactions. On the other hand, material degradation depends on the production of osteoclasts by macrophages in the immune system, and high biocompatibility reduces the chance for macrophages to recognize it. For instance, macrophages find it difficult to capture calcium phosphate materials (Gisep et al., 2003). Achieving a balance between material degradation rate and osteogenic effect remains an area for further research. Our qPCR analysis aimed to provide a molecular-level understanding of osteogenic differentiation. However, we acknowledge several limitations. Firstly, the reference genes used may not adequately normalize the results, potentially leading to biased interpretations. Secondly, the markers chosen primarily focus on osteogenesis and do not include cartilage formation markers, which are important for indicating the type of bone formation (endochondral or intramembranous) and excluding potential tumor development. These limitations highlight the need for future studies to incorporate a broader range of reference genes and markers to ensure more reliable and comprehensive results.

## 5 Conclusion

In summary, this study successfully prepared a new bone scaffold material of CFBB/Zn^2+^/CS (β-TCP-based) using the trabecular bone of the fetal bovine femur as the raw material to form CFBB first, then modified with Zn^2+^ and CS in two steps. The results demonstrate that CFBB/Zn^2+^/CS exhibits good biocompatibility and osteogenic capacity at the cellular level and in a rabbit model of mandibular defect. In the future, it is necessary to further evaluate its osteogenic capacity in various large animals, such as beagle dogs, pigs, and even rhesus monkeys, making it a promising candidate for clinical applications and contributing to the comprehensive utilization of biological waste.

## Data Availability

The raw data supporting the conclusions of this article will be made available by the authors, without undue reservation.

## Supplementary material

SUPPLEMENTARY FIGURE S1 Establishment of the rabbit jaw defect model and material implantation. **(A)** Skin preparation, disinfection; **(B)** skin incision; **(C)** after bone defect preparation; **(D)** implantation of calcined bone blocks; e: After wound closure with suture.

## References

[B1] Akay G. Birch M. Bokhari M. (2004). Microcellular PolyHIPE polymer supports osteoblast growth and bone formation *in vitro* . Biomaterials 25 (18), 3991–4000. 10.1016/j.biomaterials.2003.10.086 15046889

[B2] Amid R. Kheiri A. Kheiri L. Kadkhodazadeh M. Ekhlasmandkermani M. (2021). Structural and chemical features of xenograft bone substitutes: a systematic review of *in vitro* studies. Biotechnol. Appl. Biochem. 68 (6), 1432–1452. 10.1002/bab.2065 33135215

[B3] ASTM International (2017). Astm F756-17: standard practice for assessment of hemolytic properties of materials. American Society for Testing and Materials.

[B4] Bad M. Hesaraki S. Zamanian A. (2014). Mechanical properties and *in vitro* cellular behavior of zinc-containing nano-bioactive glass doped biphasic calcium phosphate bone substitutes. J. Mater Sci. Mater Med. 25 (1), 185–197. 10.1007/s10856-013-5062-7 24101184

[B5] Bandyopadhyay A. Withey E. Moore J. Bose S. (2007). Influence of ZnO doping in calcium phosphate ceramics. Mater. Sci. Eng. C 27 (1), 14–17. 10.1016/j.msec.2005.11.004

[B6] Beringer L. Kiechel M. Komiya Y. Donius A. Habas R. Wegst U. (2015). Osteoblast biocompatibility of novel chitosan crosslinker, hexamethylene-1,6-diaminocarboxysulfonate. J. Biomed. Mater Res. A 103 (9), 3026–3033. 10.1002/jbm.a.35438 25689675

[B7] Bin B. Seo J. Kim S. (2018). Function, structure, and transport aspects of ZIP and ZnT zinc transporters in immune cells. J. Immunol. Res. 2018, 1–9. 10.1155/2018/9365747 PMC6189677 30370308

[B8] Bohner M. Baroud G. Bernstein A. Döbelin N. Galea L. Hesse B. (2017). Characterization and distribution of mechanically competent mineralized tissue in micropores of β-tricalcium phosphate bone substitutes. Mater. Today 20 (3), 106–115. 10.1016/j.mattod.2017.02.002

[B9] Bohner M. Santoni B. Döbelin N. (2020). β-tricalcium phosphate for bone substitution: synthesis and properties. Acta Biomater. 113, 23–41. 10.1016/j.actbio.2020.06.022 32565369

[B10] Campion C. Chander C. Buckland T. Hing K. (2011). Increasing strut porosity in silicate-substituted calcium-phosphate bone graft substitutes enhances osteogenesis. J. Biomed. Mater Res. B Appl. Biomater. 97 (2), 245–254. 10.1002/jbm.b.31807 21384544

[B11] Deng F. Bu Z. Hu H. Huang X. Liu Z. Ning C. (2022). Bioadaptable bone regeneration of Zn-containing silicocarnotite bioceramics with moderate biodegradation and antibacterial activity. Appl. Mater Today 27, 101433. 10.1016/j.apmt.2022.101433

[B12] Ding P. Huang K. Li G. Zeng W. (2007). Mechanisms and kinetics of chelating reaction between novel chitosan derivatives and Zn(II). J. Hazard Mater 146, 58–64. 10.1016/j.jhazmat.2006.11.061 17244519

[B13] Elkholy S. Yahia S. Awad M. Elmessiery M. (2018). *In vivo* evaluation of β‐CS/n‐HA with different physical properties as a new bone graft material. Clin. Implant Dent. Relat. Res. 20 (3), 416–423. 10.1111/cid.12599 29479806

[B14] Feng P. Luo Y. Ke C. Qiu H. Wang W. Zhu Y. (2021). Chitosan-based functional materials for skin wound repair: mechanisms and applications. Front. Bioeng. Biotechnol. 9, 650598. 10.3389/fbioe.2021.650598 33681176 PMC7931995

[B15] Feng P. Wei P. Shuai C. Peng S. (2014). Characterization of mechanical and biological properties of 3-D scaffolds reinforced with zinc oxide for bone tissue engineering. PLoS One 9 (1), e87755. 10.1371/journal.pone.0087755 24498185 PMC3909231

[B16] Giorgio I. Spagnuolo M. Andreaus U. Scerrato D. Bersani A. (2021). In-depth gaze at the astonishing mechanical behavior of bone: a review for designing bio-inspired hierarchical metamaterials. Math. Mech. Solids 26 (7), 1074–1103. 10.1177/1081286520978516

[B17] Gisep A. Wieling R. Bohner M. Matter S. Schneider E. Rahn B. (2003). Resorption patterns of calcium-phosphate cements in bone. J. Biomed. Mater Res. A 66 (3), 532–540. 10.1002/jbm.a.10593 12918036

[B18] Hadley K. Newman S. Hunt J. (2010). Dietary zinc reduces osteoclast resorption activities and increases markers of osteoblast differentiation, matrix maturation, and mineralization in the long bones of growing rats. J. Nutr. Biochem. 21 (4), 297–303. 10.1016/j.jnutbio.2009.01.002 19369052

[B19] Han K. Sathiyaseelan A. Saravanakumar K. Wang M. (2022). Wound healing efficacy of biocompatible hydroxyapatite from bovine bone waste for bone tissue engineering application. J. Environ. Chem. Eng. 10 (1), 106888. 10.1016/j.jece.2021.106888

[B20] Hannink G. Arts J. B. (2011). Porosity and mechanical strength of bone substitutes: what is optimal for bone regeneration? Injury 42, S22–S25. 10.1016/j.injury.2011.06.008 21714966

[B21] Hing K. Revell P. Smith N. Buckland T. (2006). Effect of silicon level on rate, quality and progression of bone healing within silicate-substituted porous hydroxyapatite scaffolds. Biomaterials 27 (29), 5014–5026. 10.1016/j.biomaterials.2006.05.039 16790272

[B22] Hing K. Wilson L. Buckland T. (2007). Comparative performance of three ceramic bone graft substitutes. Spine J. 7 (4), 475–490. 10.1016/j.spinee.2006.07.017 17630146

[B23] Holzwarth J. Ma P. (2011). Biomimetic nanofibrous scaffolds for bone tissue engineering. Biomaterials 32 (36), 9622–9629. 10.1016/j.biomaterials.2011.09.009 21944829 PMC3195926

[B24] Hossain M. Uddin M. Sarkar S. Ahmed S. (2022). Crystallographic dependency of waste cow bone, hydroxyapatite, and β-tricalcium phosphate for biomedical application. J. Saudi Chem. Soc. 26 (6), 101559. 10.1016/j.jscs.2022.101559

[B25] Ito A. Senda K. Sogo Y. Oyane A. Yamazaki A. LeGeros R. (2006). Dissolution rate of zinc-containing β-tricalcium phosphate ceramics. Biomed. Mater. 1 (3), 134–139. 10.1088/1748-6041/1/3/007 18458394

[B26] Jianye Z. Rui M. Wen S. Zhanhai Y. Zhiqiang L. Xiaofen Z. (2017). Preparation, Characterization and cytocompatibility of calcined bone Blocks from adult and fetal cattle. J. Stomatology 33 (5), 475. 10.13701/j.cnki.kqyxyj.2017.05.003

[B27] Jodati H. Yılmaz B. Evis Z. (2020). A review of bioceramic porous scaffolds for hard tissue applications: effects of structural features. Ceram. Int. 46 (10), 15725–15739. 10.1016/j.ceramint.2020.03.192

[B28] Karageorgiou V. Kaplan D. (2005). Porosity of 3D biomaterial scaffolds and osteogenesis. Biomaterials 26 (27), 5474–5491. 10.1016/j.biomaterials.2005.02.002 15860204

[B29] Klincumhom N. Lorthongpanich C. Thumanu K. Septham P. Phomyu W. Issaragrisil S. (2022). Intermittent compressive force regulates human periodontal ligament cell behavior via yes-associated protein. Heliyon 8 (10), e10845. 10.1016/j.heliyon.2022.e10845 36247165 PMC9561743

[B30] Kong M. Chen X. Xing K. Park H. (2010). Antimicrobial properties of chitosan and mode of action: a state of the art review. Int. J. Food Microbiol. 144 (1), 51–63. 10.1016/j.ijfoodmicro.2010.09.012 20951455

[B31] Kuboki Y. Jin Q. Kikuchi M. Mamood J. Takita H. (2002). Geometry of artificial ECM: sizes of pores controlling phenotype expression in BMP-induced osteogenesis and chondrogenesis. Connect. Tissue Res. 43 (2-3), 529–534. 10.1080/03008200290001104 12489210

[B32] Kuboki Y. Jin Q. Takita H. (2001). Geometry of carriers controlling phenotypic expression in BMP-induced osteogenesis and chondrogenesis. J. Bone Jt. Surg. Am. 83 (1), 105–115. 10.2106/00004623-200100002-00005 11314788

[B33] Lapshin R. Alekhin A. Kirilenko A. Odintsov S. Krotkov V. (2010). Vacuum ultraviolet smoothing of nanometer-scale asperities of poly(methyl methacrylate) surface. J. Surf. Investigation 4 (1), 1–11. 10.1134/S1027451010010015

[B34] Li N. Bai R. (2005). Copper adsorption on chitosan-cellulose hydrogel beads: behaviors and mechanisms. Sep. Purif. Technol. 42 (3), 237–247. 10.1016/j.seppur.2004.08.002

[B35] Liu Y. Yan F. Yang W. Lu X. Wang W. (2013). Effects of zinc transporter on differentiation of bone marrow mesenchymal stem cells to osteoblasts. Biol. Trace Elem. Res. 154, 234–243. 10.1007/s12011-013-9683-y 23775599

[B36] Martyniak K. Wei F. Ballesteros A. Meckmongkol T. Calder A. Gilbertson T. (2021). Do polyunsaturated fatty acids protect against bone loss in our aging and osteoporotic population? Bone 143, 115736. 10.1016/j.bone.2020.115736 33171312

[B37] Maruf D. Ghosh Y. Xin H. Cheng K. Mukherjee P. Crook J. (2022). Hydrogel: a potential material for bone tissue engineering repairing the segmental mandibular defect. Polymers 14 (19), 4186. 10.3390/polym14194186 36236133 PMC9571534

[B38] Maruf D. Parthasarathi K. Cheng K. Mukherjee P. McKenzie D. Crook J. (2023). Current and future perspectives on biomaterials for segmental mandibular defect repair. Int. J. Polym. Mater. Polym. Biomaterials 72 (9), 725–737. 10.1080/00914037.2022.2052729

[B39] Monmaturapoj N. Uanlee T. Nampuksa K. Kasiwat A. Makornpan C. (2022). Preparation and properties of porous biphasic calcium phosphate/bioactive glass composite scaffolds for biomedical applications. Mater Today Commun. 33, 104993. 10.1016/J.MTCOMM.2022.104993

[B40] Pramanik S. Ataollahi F. Pingguan-Murphy B. Oshkour A. Osman N. (2015). *In vitro* study of surface modified poly(ethylene glycol)-impregnated sintered bovine bone scaffolds on human fibroblast cells. Sci. Rep. 5 (1), 9806–9811. 10.1038/srep09806 25950377 PMC4423443

[B41] Roy M. Fielding G. Bandyopadhyay A. Bose S. (2013). Effects of zinc and strontium substitution in tricalcium phosphate on osteoclast differentiation and resorption. Biomater. Sci. 1 (1), 74–82. 10.1039/c2bm00012a PMC3825406 24244866

[B42] Saravanan S. Leena R. Selvamurugan N. (2016). Chitosan based biocomposite scaffolds for bone tissue engineering. Int. J. Biol. Macromol. 93, 1354–1365. 10.1016/j.ijbiomac.2016.01.112 26845481

[B43] Saravanan S. Nethala S. Pattnaik S. Tripathi A. Moorthi A. Selvamurugan N. (2011). Preparation, characterization and antimicrobial activity of a bio-composite scaffold containing chitosan/nano-hydroxyapatite/nano-silver for bone tissue engineering. Int. J. Biol. Macromol. 49 (2), 188–193. 10.1016/j.ijbiomac.2011.04.010 21549747

[B44] Sharma G. Merz K. (2021). Formation of the metal-binding core of the ZRT/IRT-like protein (ZIP) family zinc transporter. Biochemistry 60 (36), 2727–2738. 10.1021/acs.biochem.1c00415 34455776 PMC9002132

[B45] Singh B. Veeresh V. Mallick S. Jain Y. Sinha S. Rastogi A. (2019). Design and evaluation of chitosan/chondroitin sulfate/nano-bioglass based composite scaffold for bone tissue engineering. Int. J. Biol. Macromol. 133, 817–830. 10.1016/j.ijbiomac.2019.04.107 31002908

[B46] Tan M. Shao P. Friedhuber A. Moorst M. Elahy M. Indumathy S. (2014). The potential role of free chitosan in bone trauma and bone cancer management. Biomaterials 35 (27), 7828–7838. 10.1016/j.biomaterials.2014.05.087 24947230

[B47] Tan W. Zhang J. Mi Y. Dong F. Li Q. Guo Z. (2020). Enhanced antifungal activity of novel cationic chitosan derivative bearing triphenylphosphonium salt via azide-alkyne click reaction. Int. J. Biol. Macromol. 165, 1765–1772. 10.1016/j.ijbiomac.2020.10.019 33031850

[B48] Teixeira S. Fernandes H. Leusink A. Van Blitterswijk C. Ferraz M. Monteiro F. (2010). *In vivo* evaluation of highly macroporous ceramic scaffolds for bone tissue engineering. J. Biomed. Mater Res. A 93 (2), 567–575. 10.1002/jbm.a.32532 19591232

[B49] Venkatesan J. Kim S. (2010). Chitosan composites for bone tissue engineering - an overview. Mar. Drugs 8 (8), 2252–2266. 10.3390/md8082252 20948907 PMC2953403

[B50] Wang Z. Wang W. Zhang X. Cao F. Zhang T. Bhakta D. (2022). Modulation of osteogenesis and angiogenesis activities based on ionic release from Zn–Mg alloys. Materials 15 (20), 7117. 10.3390/ma15207117 36295204 PMC9608845

[B51] Won J. Rezk A. Su K. Park H. Chun S. Kim B. (2022). Marine plankton exoskeleton-derived honeycombed hydroxyapatite bone granule for bone tissue engineering. Mater Des. 224, 111372. 10.1016/j.matdes.2022.111372

[B52] Wu L. Zhao X. He B. Jiang J. Xie X. Liu L. (2016). The possible roles of biological bone constructed with peripheral blood derived EPCs and BMSCs in osteogenesis and angiogenesis. Biomed. Res. Int. 2016, 1–11. 10.1155/2016/8168943 PMC4852345 27195296

[B53] Xue W. Dahlquist K. Banerjee A. Bandyopadhyay A. Bose S. (2008). Synthesis and characterization of tricalcium phosphate with Zn and Mg based dopants. Mater. Med. 19, 2669–2677. 10.1007/s10856-008-3395-4 18270806

[B54] Yang Y. Yu F. Zhang H. Dong Y. Qiu Y. Jiao Y. (2018). Physicochemical properties and cytotoxicity of an experimental resin-based pulp capping material containing the quaternary ammonium salt and portland cement. Int. Endod. J. 51 (1), 26–40. 10.1111/iej.12777 28375561

[B55] Yin X. Yang C. Wang Z. Zhang Y. Li Y. Weng J. (2021). Alginate/chitosan modified immunomodulatory titanium implants for promoting osteogenesis *in vitro* and *in vivo* . Mater. Sci. Eng. C 124, 112087. 10.1016/j.msec.2021.112087 33947577

[B56] Yu Z. Xiao C. Huang Y. Chen M. Wei W. Yang X. (2018). Enhanced bioactivity and osteoinductivity of carboxymethyl chitosan/nanohydroxyapatite/graphene oxide nanocomposites. RSC Adv. 8 (32), 17860–17877. 10.1039/c8ra00383a 35542061 PMC9080497

[B57] Yuan H. Fernandes H. Habibovic P. de Boer J. Barradas A. de Ruiter A. (2010). Osteoinductive ceramics as a synthetic alternative to autologous bone grafting. Proc. Natl. Acad. Sci. U. S. A. 107 (31), 13614–13619. 10.1073/pnas.1003600107 20643969 PMC2922269

[B58] Yusa K. Yamamoto O. Fukuda M. Koyota S. Koizumi Y. Sugiyama T. (2011). *In vitro* prominent bone regeneration by release zinc ion from Zn-modified implant. Biochem. Biophys. Res. Commun. 412 (2), 273–278. 10.1016/j.bbrc.2011.07.082 21820411

[B59] Zadpoor A. (2015). Bone tissue regeneration: the role of scaffold geometry. Biomater. Sci. 3 (2), 231–245. 10.1039/C4BM00291A 26218114

