# Supplementary figures and images for: Zinc and chitosan-enhanced β-tricalcium phosphate from calcined fetal bovine bone for mandible reconstruction
Source: Front Bioeng Biotechnol. 2024 Sep 27;12:1355493. doi: 10.3389/fbioe.2024.1355493 (PMC11472181; doi:10.3389/fbioe.2024.1355493)

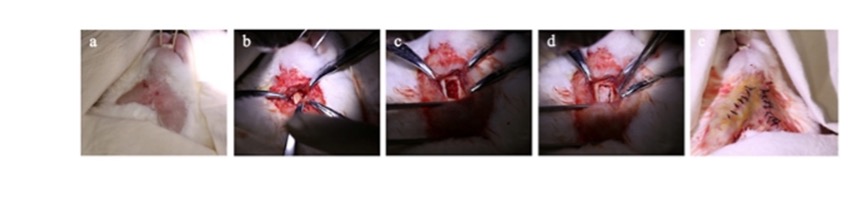

Supplement: Supplementary file 1 [file Image1.JPEG]
